# A Conserved Role for the *NAM/miR164* Developmental Module Reveals a Common Mechanism Underlying Carpel Margin Fusion in Monocarpous and Syncarpous Eurosids

**DOI:** 10.3389/fpls.2015.01239

**Published:** 2016-01-13

**Authors:** Aurélie C. M. Vialette-Guiraud, Aurélie Chauvet, Juliana Gutierrez-Mazariegos, Alexis Eschstruth, Pascal Ratet, Charles P. Scutt

**Affiliations:** ^1^Laboratoire de Reproduction et Développement des Plantes, UMR 5667, Centre National de la Recherche Scientifique – Institut National de la Recherche Agronomique – Université de Lyon, Ecole Normale Supérieure de LyonLyon, France; ^2^Institute of Plant Sciences Paris-Saclay, Centre National de la Recherche Scientifique – Institut National de la Recherche Agronomique – Université de Paris SudOrsay, France

**Keywords:** *Arabidopsis thaliana*, *Medicago truncatula*, *CUP SHAPED COTYLEDON*, *NO APICAL MERISTEM*, *miR164*, gynoecium, carpel, syncarpy

## Abstract

The majority of angiosperms are syncarpous- their gynoecium is composed of two or more fused carpels. In *Arabidopsis thaliana*, this fusion is regulated through the balance of expression between *CUP SHAPED COTYLEDON (CUC)* genes, which are orthologs of the *Petunia hybrida* transcription factor *NO APICAL MERISTEM (NAM)*, and their post-transcriptional regulator *miR164*. Accordingly, the expression of a *miR164*-insensitive form of *A. thaliana CUC2* causes a radical breakdown of carpel fusion. Here, we investigate the role of the *NAM/miR164* genetic module in carpel closure in monocarpous plants. We show that the disruption of this module in monocarpous flowers of *A. thaliana aux1-22* mutants causes a failure of carpel closure, similar to the failure of carpel fusion observed in the wild-type genetic background. This observation suggested that closely related mechanisms may bring about carpel closure and carpel fusion, at least in *A. thaliana.* We therefore tested whether these mechanisms were conserved in a eurosid species that is monocarpous in its wild-type form. We observed that expression of *MtNAM*, the *NAM* ortholog in the monocarpous eurosid *Medicago truncatula*, decreases during carpel margin fusion, suggesting a role for the *NAM/miR164* module in this process. We transformed *M. truncatula* with a *miR164*-resistant form of *MtNAM* and observed, among other phenotypes, incomplete carpel closure in the resulting transformants. These data confirm the underlying mechanistic similarity between carpel closure and carpel fusion which we observed in *A. thaliana.* Our observations suggest that the role of the *NAM/miR164* module in the fusion of carpel margins has been conserved at least since the most recent common ancestor of the eurosid clade, and open the possibility that a similar mechanism may have been responsible for carpel closure at much earlier stages of angiosperm evolution. We combine our results with studies of early diverging angiosperms to speculate on the role of the *NAM/miR164* module in the origin and further evolution of the angiosperm carpel.

## Introduction

The female whorl, or gynoecium, of the angiosperm flower consists of one or more carpels which enclose the ovules. In apocarpous gynoecia, the carpels remain separate throughout development, while in syncarpous gynoecia, they are fused together, either from their inception (congenital fusion), or from a later developmental stage (post-genital fusion). If only one carpel is produced per flower, the gynoecium is termed monocarpous. Carpels in apocarpous or monocarpous gynoecia may emerge from the floral meristem with their margins already fused together, in which case they are described as ascidiate (bottle-shaped), or may emerge with unfused margins that subsequently fuse by folding, in which case they are described as plicate.

Syncarpy is believed to confer several selective advantages over apocarpy, including a larger landing platform for pollinating insects, a compitum (a common intersection in the route for pollen tube growth), and larger fruits with more sophisticated mechanisms for seed dispersal. Mapping of character states onto angiosperm phylogeny indicates that syncarpy has arisen at least 17 times in the angiosperms, while the evolution of apocarpy from syncarpy is much less frequent (Armbruster et al., 2002).

The model angiosperm *Arabidopsis thaliana* possesses a syncarpous gynoecium of two congenitally fused carpels. These organs emerge from the center of the floral meristem as a single dome of cells, within which a central slot-like cavity forms as the gynoecium begins to elongate (Smyth et al., 1990). The positions of the carpel margins within the gynoecium wall only become apparent at a later stage, when this structure undergoes differentiation into valve and abaxial replum tissues. Meristematic activity from the abaxial replum then generates the adaxial replum, or septum, which grows inward to divide the ovary into two chambers. Ovule primordia develop from parietal placentae which form along the carpel margins within each chamber of the ovary.

In contrast to *A. thaliana*, the model angiosperm *Medicago truncatula* possesses a plicate, monocarpous gynoecium (Benlloch et al., 2003). At an early stage of *M. truncatula* flower development, the gynoecial primordium becomes crescent-shaped and its margins then fuse together to enclose the single chamber of the ovary. Ovule primordia in *M. truncatula* form from a parietal placenta that develops along the fused carpel margin.

Carpel fusion in *A. thaliana* is regulated by a genetic module, generically termed here the *NAM/miR164* module, which consists of a subset of *NAC*-family (*NAC* for *NAM, ATAF* and CUC; Aida et al., 1997) transcription factors and their post-transcriptional regulator *miR164* (Mallory et al., 2004). In *A. thaliana*, the *NAC* genes involved in this module are *CUP-SHAPED COTYLEDON1 (CUC1) and CUC2* (Aida et al., 1997), which are orthologs of the single gene *NO APICAL MERISTEM (NAM)* from *Petunia hybrida* (Souer et al., 1996). Loss of *miR164* function through mutations to all three *MIR164* paralogs in *A. thaliana* (Sieber et al., 2007), or genetic transformation of *A. thaliana* with a *miR164*-resistant version of *CUC2* (*CUC2g-m4*; Nikovics et al., 2006), results in a breakdown of carpel fusion. Accordingly, in *miR164* triple mutants or *CUC2g-m4* transformants, the two carpels of the *A. thaliana* gynoecium emerge separately and remain unfused and open throughout development.

In addition to their role in carpel development, studies of *NAM* orthologs in eudicots show these factors to be involved in meristem formation and cotyledon development (Souer et al., 1996; Aida et al., 1997, 1999; Takada et al., 2001; Weir et al., 2004), leaf development (Ishida et al., 2000; Nikovics et al., 2006; Blein et al., 2008), ovule development (Galbiati et al., 2013; Kamiuchi et al., 2014) and phyllotaxy (Peaucelle et al., 2007). These transcription factors are expressed at organ margins and tissue boundaries, and their down-regulation by *miR164* consequently facilitates organ outgrowth and/or developmental fusion. The action of the *NAM/miR164* module in the *A. thaliana* leaf margin has been modeled and found to generate, via effects on the auxin eﬄux carrier PINFORMED1 (PIN1), an alternating series of auxin maxima and minima that, respectively, generate regions of higher and lower marginal growth (Bilsborough et al., 2011).

In this work, we hypothesized that the role of the *NAM/miR164* module in syncarpous fusion in *A. thaliana* might reflect a more general role in the fusion of carpel margins in angiosperms. Consequently, we tested the role of this module in the closure of monocarpous gynoecia produced both in *A. thaliana aux1-22* mutants, which are null mutants of the *AUX1* auxin influx transporter (Bennett et al., 1996) and in a wild-type genetic background of the distantly related eurosid *M. truncatula*. From the results of these experiments, we conclude that the *NAM/miR164* module has conserved a role in carpel margin fusion, at least since the most recent common ancestor (MRCA) of living eurosids. A detailed comparison of gene expression patterns suggests that fine-tuning of the *NAM/miR164* module may regulate species–specific differences in the timing of carpel margin fusion. Accordingly, we discuss the possibility that the activity of the *NAM/miR164* module may be conserved in carpel development throughout the angiosperms, while subtle modulations to this mechanism may determine the distinction between congenital and post-genital carpel margin fusion events in specific angiosperm groups. We further speculate on mechanisms acting upstream of the *NAM/miR164* module that may have contributed to the origin of the carpel in the first flowering plants.

## Materials and Methods

### Plant Cultivation

*Arabidopsis thaliana* plants were grown from seed on peat-based compost in growth chambers at a daytime temperature of ∼21°C and ∼55% relative humidity (RH). Plants were initially grown under 8/16 h day/night cycles generated using fluorescent lighting consisting of equal numbers of “cool daylight” (Osram Lumilux L36W/865) and “warm white” (Osram Lumilux L36W/830) lamps, giving a total photon flux at bench level of 170 μmol.m^-2^.s^-1^. To induce flowering, plants were transferred to long days (16/8 h day/night cycles) under otherwise similar conditions.

*Medicago truncatula* plants were grown from seed on peat-based compost in a greenhouse at a daytime temperature of ∼22.5°C and 40–60% RH under natural daylight, extended to 16 h daylength using sodium lamps, as necessary.

### Vector Construction

*MtNAM* (MTR_2g078700; Cheng et al., 2012) was initially isolated by radioisotopic screening of an *M. truncatula* bacterial artificial chromosome (BAC) library (Nam et al., 1999). A 1.2-kb fragment containing the *miR164*-binding site of *MtNAM* was released from a sub-cloned BAC DNA fragment by cleavage with *Sst*I and re-ligated into the *pGEM T-Easy* vector. The resulting plasmid was subjected to oligonucleotide-directed site-specific mutagenesis following the method of Kirsch and Joly (1998), using the sense- and antisense-strand oligonucleotides 5′-GAGCACGTGTCCTGTTTtagtACAACATCTACAACATC and 5′-GATGTTGTAGATGTTGTactaAAACAGGACACGTGCTC, respectively. These oligonucleotides generate the same four-base mismatch (shown above in lower case) present in the *miR164*-binding site of *CUC2g-m4* (Nikovics et al., 2006). Mutagenised and wild-type versions of a *MtNAM* genomic sequence of 9883 bp, from an *Eco*RI site situated 6437 bp upstream of the *MtNAM* initiation codon to an *Nco*I site situated 2151 bp downstream of its termination codon, were then inserted by ligation between unique *Eco*R1 and *Not*1 sites situated between the Left and Right T-DNA borders of the *pGREEN II-NosHyg* plant transformation vector, thereby generating the plasmids *MtNAMg-m4* and *MtNAMg-wt*, respectively.

### Plant Transformation

*Arabidopsis thaliana aux1-22* mutants (null mutants of *AUX1*; Bennett et al., 1996) were transformed by the “floral dip” method (Clough and Bent, 1998) using the *CUC2g-wt* and *CUC2g-m4* constructs of Nikovics et al. (2006) in *Agrobacterium tumefaciens* strain GV3101 harboring the plasmids *pMP90* (Koncz and Schell, 1986) and *pSOUP* (Hellens et al., 2000). Transformants were selected on plant agar containing 50 μg/mL kanamycin.

*MtNAMg-wt* and *MtNAMg-m4* constructs were introduced into *A. tumefaciens* GV3101, as described above, and used to transform *M. truncatula* R108 leaf disks by the protocol of Cosson et al. (2015), in which transgenic calli were selected on media containing 30 μg/mL hygromycin.

### *In Situ* Hybridization

Double-stranded cDNAs representing the full-length coding sequences of *A. thaliana CUC1* and *CUC2* and of *M. truncatula MtNAM* were generated by reverse-transcriptase PCR, incorporating a T7-RNA-Polymerase promoter sequence in the reverse primer. Digoxgenin-labeled riboprobes were prepared from these templates using T7 RNA-polymerase and these were then purified and used in *in situ* hybridizations to sections of fixed floral buds embedded in Paraplast Xtra (Leica-Surgipath), as described by Vialette-Guiraud et al. (2011b). Gene expression patterns were observed and photographed under bright field illumination using a Leica Axio Imager M2 inverted microscope fitted with a Leica AxioCam MRc digital camera.

### Phenotypic Observations

Flower buds were dissected, observed, and photographed using a Leica MZ12 dissecting microscope fitted with an AxioCam ICc5 digital camera. Carpel anatomy was revealed in transverse sections of fixed flower buds, prepared as for *in situ* hybridization and stained with 0.05% (w/v) Toluidine Blue-0 in 0.1 M sodium phosphate buffer (pH 6.8). Scanning electron microscopy was performed on unfixed material using a Hirox 3000 bench-top environmental scanning electron microscope (SEM).

### Character State Mapping

A partial cladogram of angiosperm phylogeny was produced, based on the current consensus view of angiosperm phylogeny given by the Angiosperm Phylogeny Group III (APG III, http://www.mobot.org/MOBOT/research/APweb/; Bremer et al., 2009). Carpel fusion character states, obtained from the APG III website and from bibliographic searches, were mapped on this cladogram by maximum parsimony using MacClade4 software.

## Results

### Monocarpy in *Medicago truncatula* Arose by Reversion from Syncarpy in a Common Ancestor Shared with *Arabidopsis thaliana*

**FIGURE 1:**
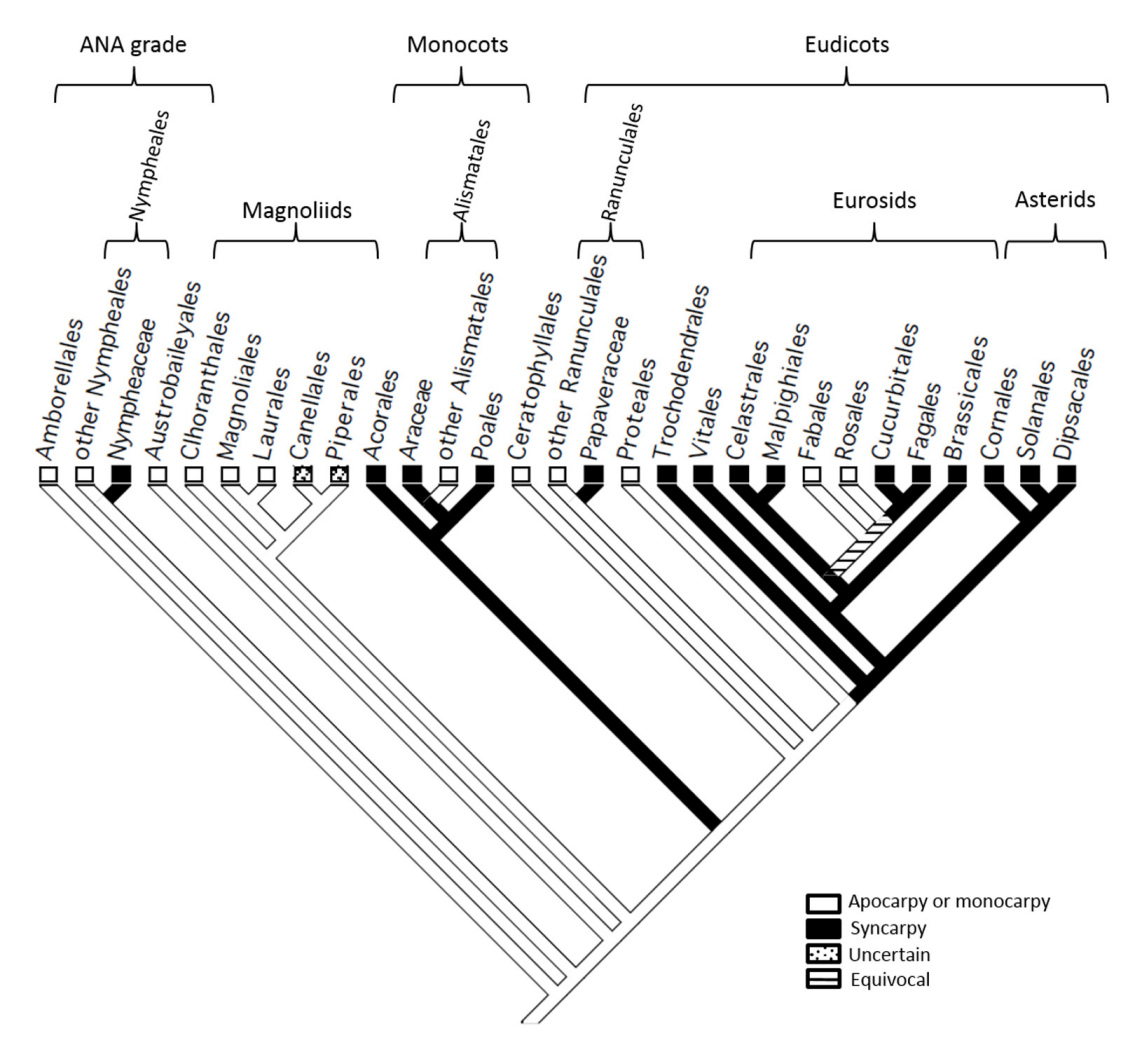
**A partial cladeogram of the angiosperms showing evolutionary transitions in carpel fusion**. Monocarpy in Fabales (including *Medicago trucatula*) is revealed in this analysis to have reverted from syncarpy present in a common ancestor shared with Brassicales (including *Arabidopsis thaliana*), Celastrales and Malpighiales.

To elucidate transitions in carpel fusion in the angiosperms, with emphasis on the model eurosids *M. truncatula* and *A. thaliana*, we mapped this character state onto a cladogram (**Figure 1**) representing the consensus view of angiosperm phylogeny (Bremer et al., 2009). This analysis confirms the findings of earlier studies (Armbruster et al., 2002) which indicated that the MRCA of living angiosperms was apocarpous, and that syncarpy arose several times independently, including in Nymphaeaceae, monocots, Papaveraceae and a common ancestor of the rosids and asterids. Within the eurosids, our analysis indicates that monocarpy in Fabales (including *M. truncatula*), arose secondarily from syncarpy, which was present in a common ancestor shared with Brassicales (including *A. thaliana*), Celastrales and Malpighiales. By localizing transitions between apocarpy/monocarpy and syncarpy, this analysis provides a phylogenetic framework for the evolutionary interpretation of data on the molecular mechanisms involved in these processes in living angiosperms.

### The *NAM/miR164* in *Arabidopsis thaliana* Plays a Role in Both Syncarpy and the Closure of Single Carpels

As the *NAM/miR164* developmental module is necessary for carpel fusion in wild-type, syncarpous *A. thaliana* (Nikovics et al., 2006; Sieber et al., 2007), we aimed to discover whether this mechanism could also contribute to the closure of single carpels in this species. To do this, we tested whether the introduction of a *miR164*-resistant version of *CUC2* (*CUC2g-m4*) could cause a breakdown in the closure of the single carpels that are produced in *A. thaliana aux1-22* mutants (Bennett et al., 1996), as compared to control plants transformed with a wild-type construct (*CUC2g-wt*).

**FIGURE 2:**
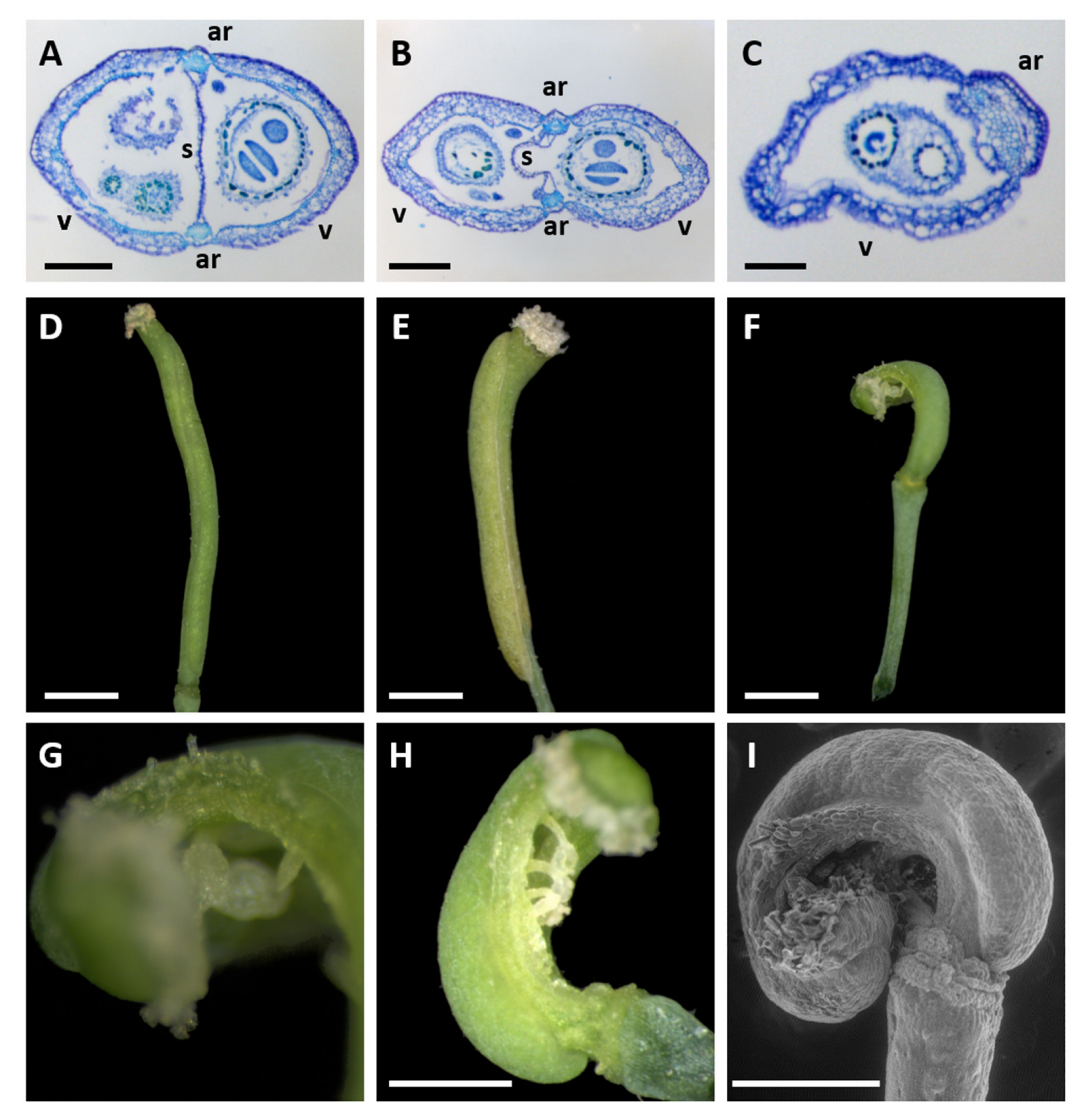
**Gynoecium morphology of *A. thaliana aux1-22* mutants transformed with *miR164*-resistant (*CUC2g-m4*) or un-mutated control (*CUC2g-wt*) constructs. (A–C)** Toluidine blue staining of transverse sections of Col-0 wild-type **(A)** and *aux1-22* mutant gynoecia composed of two fused carpels **(B)**, and one closed carpel **(C)**, respectively. **(D,E)** An untransformed *aux1-22* mutant **(D)** and an *aux1-22 CUC2g-wt* (control) transformant showing entirely closed monocarpous gynoecia. **(F–I)** *aux1-22 CUC2g-m4* transformants showing a breakdown carpel closure in monocarpous gynoecia. (**G** is a magnification of the apex of the carpel shown in **(F). I** is a scanning electron microscope image) ar, abaxial replum; s, septum (or adaxial replum); v, valve. Bars = 200 μm in **(A,B)**, 100 μm in **(C)**, and 1 mm in **(D–F,H,I)**.

Wild-type Col-0 gynoecia are syncarpous (**Figure 2A**), as are approximately 50% of gynoecia produced in *aux1-22* mutants (**Figure 2B**). The ovary wall in these gynoecia contains two valves, alternating with two abaxial repla. The monocarpous gynoecia, which are also produced in *aux1-22* mutants (**Figures 2C,D**), develop as closed structures whose ovary wall consists of only one valve and one abaxial replum (**Figure 2C**). These monocarpous gynoecia are not divided by a septum, or adaxial replum. Transformation of *aux1-22* mutants with *CUC2g-wt* produced no apparent change in the morphology of monocarpous gynoecia (**Figure 2E**). However, transformation of these mutants with *CUC2g-m4* produced a high proportion of monocarpous gynoecia that remained open to maturity (**Figures 2F–I**). In eight of 20 T1 transformants analyzed, all flowers containing two carpels showed carpel fusion defects, while all monocarpous flowers showed a complete or partial lack of carpel closure, remaining open over part or all of the valve margin. In these eight plants, carpel fusion/closure defects resulted in an almost complete loss of female fertility. Thus, disruption of the *NAM/miR164* developmental module in monocarpous mutant gynoecia of *A. thaliana* causes the failure of developmental closure in these structures in a similar manner to the disruption of carpel fusion in syncarpous, wild-type gynoecia.

### Expression of *NAM* Orthologs is Absent or Reduced During Carpel Margin Fusion in *Arabidopsis thaliana* and *Medicago truncatula*

The observation that the *NAM/miR164* module regulates developmental closure events in the gynoecium in both syncarpous and monocarpous genotypes of *A. thaliana* led us to speculate that this molecular mechanism might be widely conserved within the angiosperms. We chose *M. truncatula*, which produces in its wild-type form a single carpel in each flower, as a candidate model species in which to test this hypothesis. The MRCA between *M. truncatula* and *A. thaliana*, which is also the MRCA of the living eurosid clade (comprising Fabidae, or eurosids I and Malvidae, or eurosids II), is estimated to have lived 114–113 million years ago (MYA; Wang et al., 2009). Prior to initiating functional experiments in *M. truncatula*, we used *in situ* hybridization to examine the conservation of expression of *NAM* orthologs in flower tissues between *A. thaliana* and *M. truncatula* and thereby ascertain the likelihood that the *NAM/miR164* module might function in carpel closure in the latter species.

**FIGURE 3:**
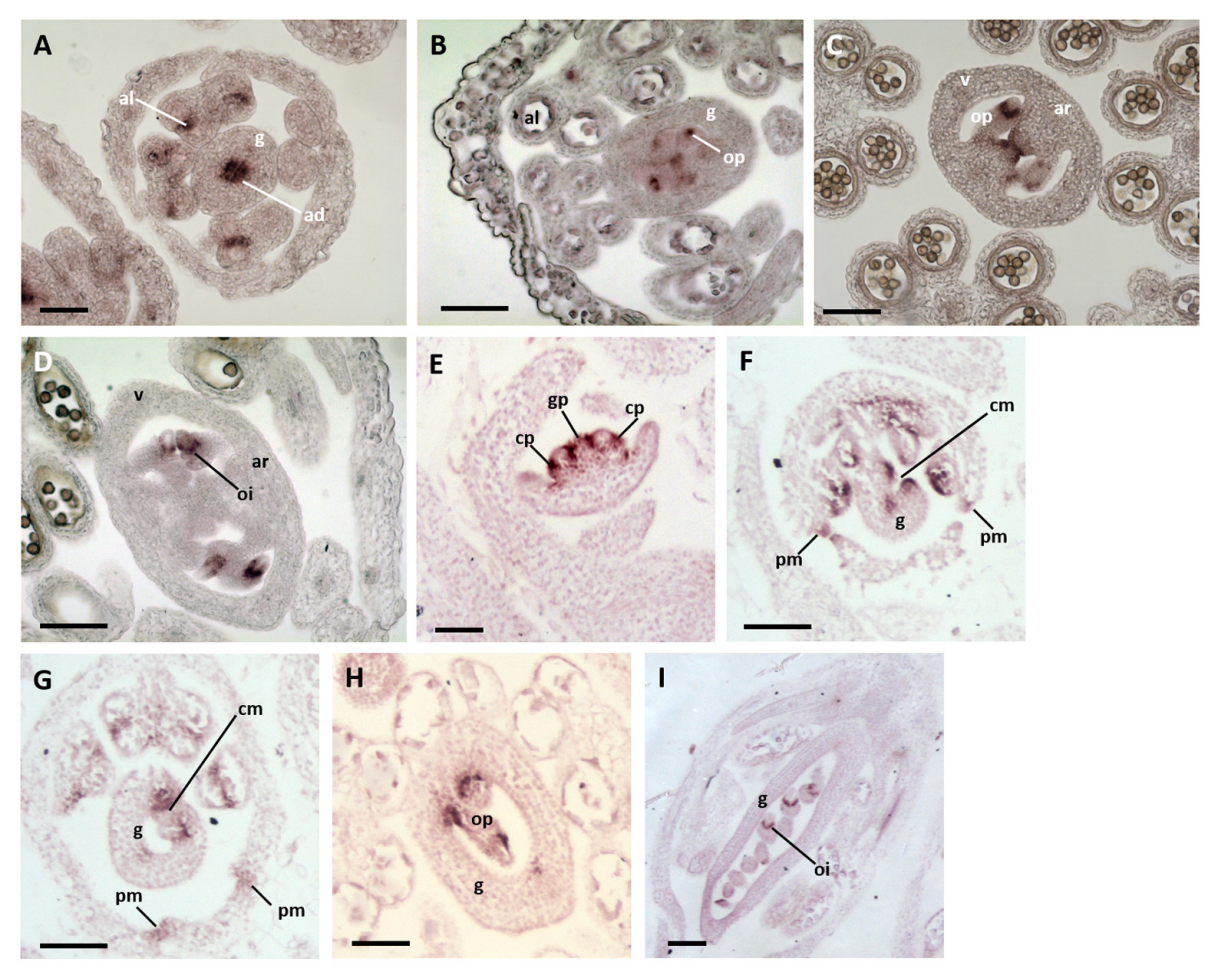
**Expression of *NAM* orthologs in *A. thaliana* and *Medicago truncatula* flower buds. (A–D)** *A. thaliana* flower buds hybridized to *CUC1* and *2* probes. **(A)** A Stage-7 bud showing *CUC2* expression in the abaxial domain of the gynoecium and anther locculi. **(B,C)** Buds at Stages 9–10 showing *CUC1* **(B)** and *CUC2* **(C)** expression in the placenta and within ovule primordia. **(D)** A bud at Stage 11 showing *CUC1* expression at the base of the expanding ovule integuments. ad, adaxial zone of the gynoecium; al, anther loculus; ar, abaxial replum; g, gynoecium; oi, ovule integuments; op, ovule primordium. Bars = 50 μm. **(E–I)** *M. truncatula* flower buds hybridized to an *MtNAM* probe. **(E)** A bud at Stages 3–4 showing *MtNAM* expression between and within organ primordia. **(F)** A Stage-7 bud showing strong *MtNAM* expression in the unfused carpel margins. **(G)** A bud at Stage 8, in which *MtNAM* expression is reduced in the fused carpel margins. **(H,I)** Later stages of flower development in which *MtNAM* expression is absent in the carpel margins, but present first in ovule primordia **(H)** and then at the base of the expanding integuments **(I)**. cm, carpel margin; cp, common primordium; g, gynoecium; gp, gynoecial primordium; oi, ovule integument; op, ovule primordium; pm, petal margin. Bars = 100 μm.

*In situ* hybridization in *A. thaliana* flowers at Stage 7 (Smyth et al., 1990), in which a central slot is beginning to form in the gynoecial cylinder, revealed the expression of *CUC2* in the adaxial domain of the gynoecium and in the loculi of the developing anthers (**Figure 3A**). Recent studies (Galbiati et al., 2013) revealed similar results for *CUC1*. Thus, no expression of either *CUC1* or *CUC2* has been detected in regions of the ovary wall destined to become the abaxial repla, or the fusion zones between these tissues and the valves. At Stage 9–10, both *CUC1* and *CUC2* were expressed in the placentae and at presumptive tissue boundaries within the elongating ovule primordia (**Figures 3B,C**). At Stage 11, *CUC1* was expressed at the base of the expanding ovule integuments (**Figure 3D**).

*In situ* hybridization in *M. truncatula* at Stages 3–4 of flower development, following the time course defined by Benlloch et al. (2003), showed *MtNAM* expression between the gynoecium primordium and the surrounding common primordia that give rise to both stamens and petals (**Figure 3E**). Signals were also detected within these common primordia (**Figure 3E**), marking the boundary between the zones destined to produce petals and stamens. At Stage 7, the gynoecium appeared crescent-shaped in transverse section and *MtNAM* was clearly expressed in the carpel margins, and at the margins of the developing free petals (**Figure 3F**). By early Stage 8, expression of *MtNAM* was observed to decline in the carpel margins (**Figure 3G**), which had, by this time, fused together to close the gynoecium. At later developmental stages, *MtNAM* expression is present in presumptive tissue boundaries in the elongating ovule primordia (**Figure 3H**) and, following this, at the base of the expanding integuments of the ovule (**Figure 3I**). Similar *NAM*-ortholog expression patterns in floral organ and ovule primordia were previously shown in another species of Fabaceae, *Pisum sativum* (Blein et al., 2008).

These expression data reveal several underlying similarities in the expression of *miR164*-regulated *NAM* orthologs between *A. thaliana* and *M. truncatula*. These orthologs are highly expressed in both species at frontiers between and within floral organs, particularly during ovule development. These data do, however, reveal a difference in *NAM* expression in the carpel margins- no such expression was detected in the presumptive abaxial repla of the gynoecial tube at early stages of *A. thaliana* flower development, whereas *NAM* expression was detected in the carpel margins of the early *M. truncatula* gynoecium. This difference may relate to the contrasting modes of congenital and post-genital carpel margin fusion in *A. thaliana* and *M. truncatula*, respectively. Despite the differences observed, we concluded that the presence of *MtNAM* expression in *M. truncatula* carpel margins suggested that the *NAM/miR164* module may be involved in the fusion of these structures, leading us to test this hypothesis experimentally.

### Expression of a *miR164*-Resistant form of *MtNAM* Leads to a Breakdown in Carpel Margin Fusion and Other Developmental Fusion Events in *Medicago truncatula* Flowers

**FIGURE 4:**
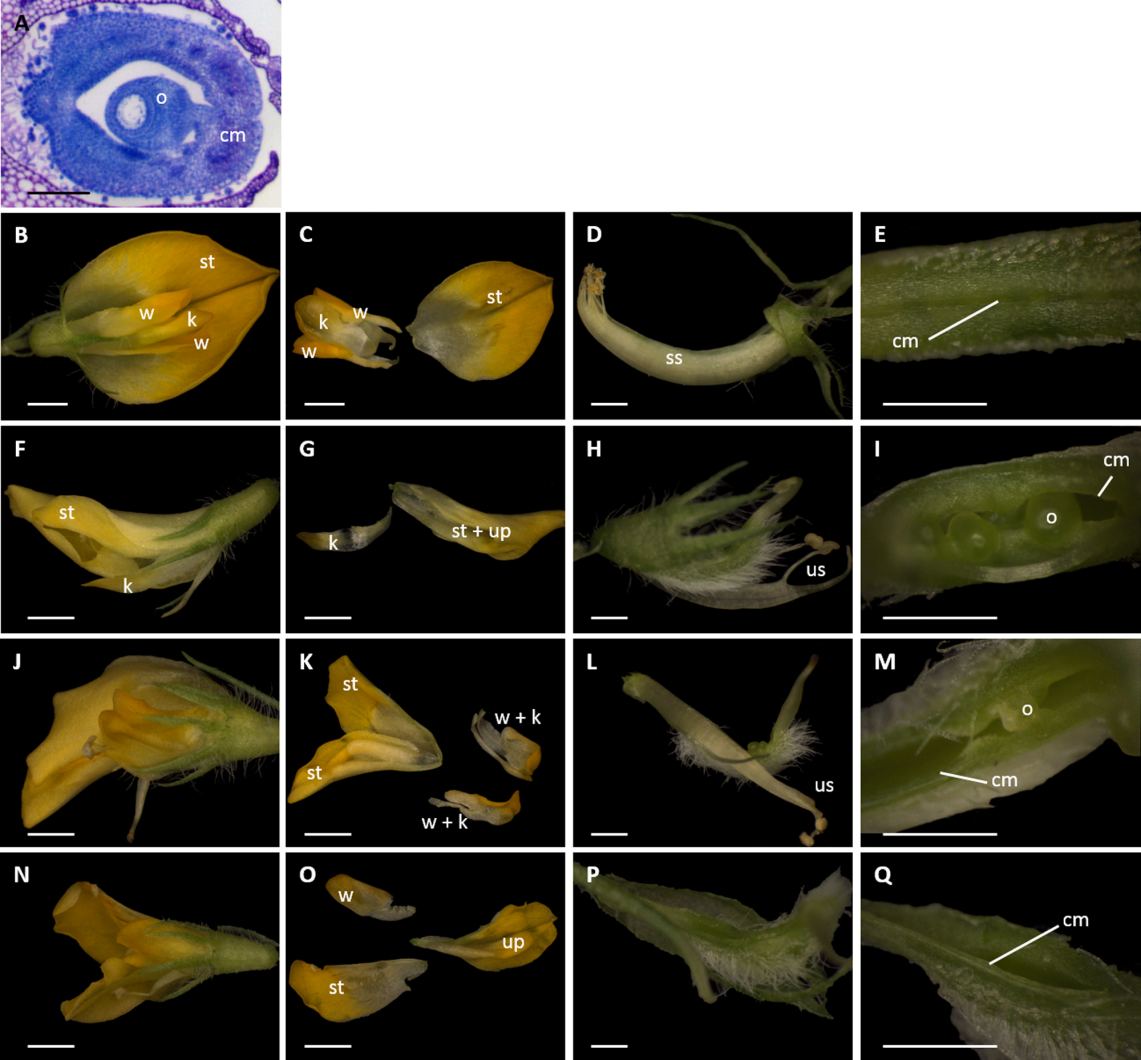
**Dissections of *M. truncatula* flowers transformed with *miR164*-resistant (*MtNAMg-m4*) or wild-type control (*MtNAMg-wt*) constructs. (A)** Transverse section of wild-type *M. truncatula* gynoecium stained with toluidine blue. **(B–E)** A typical flower of an *MtNAMg-wt* transformant showing **(B)** the intact flower, **(C)** petal morphology, **(D)** the sheath of anther filaments surrounding the gynoecium, and **(E)** the carpel margins. All structures in **(B–E)** appear identical to wild-type. **(F–I)**, **(J–M)**, and **(N–Q)** Three representative flowers from *MtNAM-m4* transformants showing **(F,J,N)** the intact flower, **(G,K,O)** petal morphology, **(H,L,P)** after removal of the perianth, and **(I,M,Q)** the carpel margins. Defects in the corolla, androecium and gynoecium are apparent, including a marked breakdown in carpel margin fusion in most flower buds (e.g., **I,M**). cm, carpel margin; k, keel petal(s); o, ovule; ss, stamen sheath; st, standard petal; up, unidentified petal(s); us, unfused stamens; w, wing petal. Bars = 100 μm in **(A)**, 1 mm in **(B,C,F,G,J,K,N,O)**, and 0.5 mm in **(D,E,H,I,L,M,P,Q)**.

To test the role of the *NAM/miR164* developmental module on carpel closure in *M. truncatula*, we produced transgenic plants expressing genomic constructs of *MtNAM* (*MtNAMg-m4* and *MtNAMg-wt*), respectively, with or without four point mutations in their predicted *miR164*-binding sites, identical to those present in the *CUC2g-m4* construct (Nikovics et al., 2006). Three independent transgenic *MtNAMg-m4* calli were generated, two of which were successfully regenerated into fertile adult plants, as was one transgenic callus containing an *MtNAMg-wt* construct (**Table 1**). Phenotypic observations were made on T2 progeny representative of one of each of these transformed lines, and on untransformed plants for comparison (**Table 2**; **Figure 4**).

**Table 1:** Results summary for transformation of *Medicago truncatula.*

Constructs	Number of transformation experiments performed	Number of trangenic calli produced (combining all experiments)	Number of plantlets regenerated	Number of T1 plants surviving to reproductive phase
*MtNAMg-wt*	2	7 (cal 1–7)	7 from cal 1	2 from cal 1
*MtNAMg-m4*	2	3 (cal 1–3)	18 from cal 1 1 from cal 2 1 from cal 3	12 from cal 1 1 from cal 2

**Table 2:** Floral phenotypes of five representative T2 *MtNAMg-m4* transformants.

T1 parent	T2 plant	Total number of flowers dissected	Number of flowers showing abnormalities in the corolla	Number of flowers showing abnormalities in the androecium		Number of flowers showing abnormalities in the gynoecium	
				Unfused stamens	Stamens absent	Slight defects in carpel fusion	Extensive defects in carpel fusion
m4 cal 1	1_1	3	3	0	0	0	0
m4 cal 1	1_2	17	6	2	0	2	7
m4 cal 1	4_1	13	11	3	0	0	4
m4 cal 1	5_1	14	5	0	0	3	2
m4 cal 1	6_3	12	12	4	5	2	8

Toluidene-blue staining was performed to highlight the ovule and the fused region of the carpel margins in the gynoecium of untransformed *M. truncatula* (**Figure 4A**). Transformation with *MtNAMg-wt* (**Figures 4B–E**) showed no effects on flower development compared to wild type *M. truncatula*. Accordingly, in *MtNAMg-wt* transformants, as in wild-type, five petals were produced, including two fused “keel” petals, two unfused “wing” petals, and a single “standard” petal (**Figures 4B,C**). As in the wild-type, all stamen filaments, with the exception of a single stamen positioned adjacent to the standard, were fused into a sheath surrounding the gynoecium (**Figure 4D**). The carpel margins of *MtNAMg-wt* transformants were also developmentally fused in the mature gynoecium, as in wild-type (**Figure 4E**).

By contrast, a range of mutant phenotypes were noted in flowers of plants transformed with the *MtNAMg-m4* construct (**Table 2**; **Figures 4F–Q**). Two standard petals were produced in some flowers (**Figure 4K**), while in others, petals with altered morphology and fusion were produced, rendering difficult their identification as standard, wing or keel petals (**Figures 4G,O**). Unfused stamens were produced in some cases (**Table 2**; **Figures 4H,L**), while stamens were absent in others (**Table 2**; **Figure 4Q**). The carpel margins remained unfused in many flowers (**Table 2**; **Figures 4I,M**), revealing the ovules within these, though a small proportion of flowers did show completely fused carpel margins (**Table 2**; **Figure 4Q**).

These data indicate a range of roles of the *NAM/miR164* developmental module in fusion events in the corolla, androecium, and gynoecium of *M. truncatula* flowers. Of particular interest to the current work, the elimination of post-transcriptional regulation of *MtNAM* in the gynoecium is shown to have a similar effect in *M. truncatula* to that shown on *aux1-22* mutants of *A. thaliana* (**Figure 2**) by disrupting the fusion of carpel margins.

## Discussion

### A Role of the *NAM/miR164* Module in the Fusion of Carpel Margins has Been Conserved at Least Since the MRCA of the Eurosids

In this study, we show that a previously characterized developmental module involving the post-transcriptional regulation of *NAM* orthologs by *miR164* is involved not only in carpel fusion in syncarpous *A. thaliana* (Nikovics et al., 2006; Sieber et al., 2007), but also in the closure of the single carpels present in two species whose lineages diverged at the base of the eurosid clade, some 114–113 MYA. The two species concerned are *A. thaliana* itself, as *aux1-22* mutants of *A. thaliana* produce single carpels, and *M. truncatula*, which is monocarpous in its wild-type form. We show that disruption of the *NAM/miR164* module in both *A. thaliana aux1-22* mutants (**Figure 2**) and a wild-type background of *M. truncatula* (**Figure 4**) produces single carpels that are no longer completely fused at their margins.

These data indicate that the *NAM/miR164* module has conserved a role in developmental fusion events between carpel margins at least since the MRCA of the eurosids. The mapping of character states onto angiosperm phylogeny (**Figure 1**) indicates that the MRCA of the eurosids was syncarpous, and we may thus conclude that the *NAM/miR164* module contributed to carpel fusion in that key ancestor, from which some 70 000 extant species are descended (Wang et al., 2009).

### The *NAM/miR164* Module Maintained its Role in Carpel Margin Fusion During a Transition from Syncarpy to Monocarpy in an Ancestor of Fabales

Character-state mapping (**Figure 1**) further indicates that the monocarpy present in Fabales (including *M. truncatula*) is a derived condition that occurred by reversion from syncarpy, present in earlier eurosids. In the present work, we show that the role of the *NAM/miR164* module in carpel margin fusion was conserved during this developmental transition. Thus, our study strongly suggests that the *NAM/miR164* module provides an underlying mechanism that is necessary for fusion events at the carpel margins of both syncarpous and monocarpous eurosids.

It is interesting to note that the *aux1-22* mutation in *A. thaliana* causes a transition from a congenitally fused gynoecium of two carpels to a closed, monocarpous gynoecium. Thus, a single loss-of-function mutation in a gene involved in auxin signaling can bring about, in *A. thaliana*, a similar type of morphological transition to that which led to monocarpy in Fabales. The genetic simplicity of this transition suggests that reversions from syncarpy to monocarpy might occur frequently in natural populations. The general trend in the angiosperms, however, is for evolutionary transitions toward syncarpous gynoecia, which are believed to confer numerous selective advantages (Armbruster et al., 2002). Thus, while the loss of syncarpy may be a genetically “easy” transition to make, the fixation of this trait in populations by natural selection may occur much less frequently.

### A Possible Role for the *NAM/miR164* Module in the Timing of Carpel Fusion

In *A. thaliana*, the gynoecium forms as a radially symmetrical cylinder that later differentiates to show the positions of the carpel margins. By contrast, the single carpel of the *M. truncatula* gynoecium is plicate, and closes post-genitally by the fusion of preexisting carpel margins. *In situ* hybridization in this work (**Figure 3**) and other studies (Galbiati et al., 2013) failed to detect any expression of *CUC1* or *CUC2* in the carpel margins of *A. thaliana*. However, *CUC2* is known to be highly expressed in the carpel margins of the unfused gynoecium at Stage 9 of flower development in *mir164abc* triple mutants (Sieber et al., 2007). Comparison of these data strongly suggests that the *NAM/miR164* expression balance in *A. thaliana* lies heavily in favor of *miR164* from the earliest stages of gynoecium development. By contrast, detectable levels of *MtNAM* were present in margin tissues at early stages of *M. truncatula* carpel development, and these levels were observed to decline at subsequent stages, as the margins fused (**Figure 3**). Thus, the different balances of *NAM* and *miR164* expression observed at very early stages of *A. thaliana* and *M. truncatula* carpel development (**Figure 3**; Galbiati et al., 2013) correlate closely with the different timings of carpel closure observed in these species (Smyth et al., 1990; Benlloch et al., 2003).

Given the role of the *NAM/miR164* module in carpel closure in both *A. thaliana* and *M. truncatula* (**Figures 2** and **4**), and the gene expression differences we have noted between the congenitally and post-genitally fused carpel margins of these two respective species, it would be interesting to compare the expression of *NAM* orthologs in a range of Fabales that show different spatial and temporal patterns of carpel closure. Candidate species for this analysis include *Acacia celastrifolia* and *Inga bella* (Paulino et al., 2014), in which the carpels include both congenitally fused (ascidiate) and later-fusing (plicate) zones, and *Amberstia nobilis* and *Caesalpina* spp. (Tucker and Kantz, 2001), in which the carpel margins remain unfused until after ovule initiation, much later than in most other Fabales. Such experiments, in quite closely related species showing marked differences in gynoecium anatomy, could provide strong correlative evidence of a role for the subtle modulation of gynoecium development by changes to the balance of the *NAM/miR164* module. Notably, the *NAM/miR164* expression balance at very early stages of carpel development may be important in determining whether carpel margins will fuse congenitally or postgenitally.

### The Role of the *NAM/miR164* Module in Carpel Evolution

As its genetic components are present in both gymnosperms and angiosperms (Axtell and Bartel, 2005; Larsson et al., 2012), the *NAM/miR164* genetic module is clearly of ancient origin in seed plants. This module is involved in leaf, carpel, and ovule development in model angiosperms (Nikovics et al., 2006; Blein et al., 2008; Galbiati et al., 2013; Goncalves et al., 2015), while expression studies in *Amborella trichopoda*, the only living representative of Amborellales (see **Figure 1**), and hence the likely sister to all other living angiosperms, suggest that its role in ovule development, at least, has been conserved from the earliest stages of angiosperm evolution (Vialette-Guiraud et al., 2011a).

Like most basally diverging angiosperms, *A. trichopoda* is apocarpous and has ascidiate carpels. Thus, in both *A. trichopoda* and *A. thaliana*, the carpel margins are congenitally fused from the earliest stages of gynoecium development, albeit in the different contexts of apocarpy and syncarpy, respectively. No expression of the *NAM* ortholog from *A. trichopoda, AtrNAM*, was observed in the early carpel wall (Vialette-Guiraud et al., 2011a), as is the case for *CUC1* and *CUC2* in *A. thaliana* (Galbiati et al., 2013; **Figure 3**). Thus, it appears reasonable to postulate that the *NAM/miR164* module operates in favor of the expression of *miR164*, and against that of *NAM* orthologs, from the earliest stages of gynoecium development in *A. trichopoda*, as it does in *A. thaliana*.

From the above observations, we hypothesize that the *NAM/miR164* module may have played a role in the fusion of carpel margins in the MRCA of the living angiosperms, as it does in present-day model angiosperms. An important test of this hypothesis will depend on the development of plant transformation strategies in basally diverging angiosperms, which would allow, for example, the transformation of *A. trichopoda* with a *miR164*-resistant form of *AtrNAM*. Comparison of early diverging angiosperm lineages strongly suggests that the first flowering plants possessed ascidiate, rather than plicate carpels (Endress and Igersheim, 2000). Accordingly, we furthermore hypothesize, based on our gene expression analyses (**Figure 3**), that the origin of plicate carpels in various later-emerging angiosperm lineages may have depended on subtle modifications to the *NAM/miR164* module that allowed a limited level of early expression of *NAM* orthologs in the carpel margins, as occurs in present-day *M. truncatula*.

Interestingly, it is known that in *A. thaliana*, the role of *CUC2* in the closure of the gynoecium apex is under indirect negative control by the bHLH transcription factor SPATULA (SPT; Nahar et al., 2012). In addition, SPT is known to play a role in carpel fusion along the entire length of the gynoecium, which is revealed in double-mutant combinations with the YABBY transcription factor *CRABS CLAW* (*crc-1 spt-2*; Alvarez and Smyth, 1999). Like the *NAM/miR164* module, it seems that SPT may have conserved its function in carpel development from the earliest stages of angiosperm evolution (Reymond et al., 2012). Thus, the establishment of negative regulation by SPT of a *miR164*-regulated *NAM* gene in a common ancestor of the angiosperms may have been a crucial step in the evolution of the closed carpel. Analysis of the pathway linking SPT, and its cofactors such as the HECATE transcription factors (Schuster et al., 2015), with the *NAM/miR164* module in model angiosperms could provide insights into this possibility, and thus potentially indicate a molecular mechanism for the enclosure of the ovule with the carpel in the first angiosperms.

## Author Contributions

AV-G performed all of the work except Medicago transformation, prepared the figures and collaborated with CS to plan and write the paper. AC assisted with *in situ* hybridizations of *Arabidopsis*. JG-M assisted with *in situ* hybridizations of Medicago. AE performed Medicago transformations. PR supervised Medicago transformations. CS supervised all work except Medicago transformation and collaborated with AV-G to plan and write the paper.

## Conflict of Interest Statement

The authors declare that the research was conducted in the absence of any commercial or financial relationships that could be construed as a potential conflict of interest.
